# Host-associated differentiation in a highly polyphagous, sexually reproducing insect herbivore

**DOI:** 10.1002/ece3.1526

**Published:** 2015-06-01

**Authors:** Josephine B Antwi, Gregory A Sword, Raul F Medina

**Affiliations:** 1 Department of Entomology, Texas A&M UniversityCollege Station, Texas; 2 Faculty of Ecology and Evolutionary Biology, Texas A&M UniversityCollege Station, Texas

**Keywords:** Amplified fragment length polymorphisms, cotton fleahopper, gene flow, host-associated differentiation, native plants

## Abstract

Insect herbivores may undergo genetic divergence on their host plants through host-associated differentiation (HAD). Much of what we know about HAD involves insect species with narrow host ranges (i.e., specialists) that spend part or all their life cycle inside their hosts, and/or reproduce asexually (e.g., parthenogenetic insects), all of which are thought to facilitate HAD. However, sexually reproducing polyphagous insects can also exhibit HAD. Few sexually reproducing insects have been tested for HAD, and when they have insects from only a handful of potential host-plant populations have been tested, making it difficult to predict how common HAD is when one considers the entire species’ host range. This question is particularly relevant when considering insect pests, as host-associated populations may differ in traits relevant to their control. Here, we tested for HAD in a cotton (*Gossypium hirsutum*) pest, the cotton fleahopper (CFH) (*Pseudatomoscelis seriatus*), a sexually reproducing, highly polyphagous hemipteran insect. A previous study detected one incidence of HAD among three of its host plants. We used Amplified fragment length polymorphism (AFLP) markers to assess HAD in CFH collected from an expanded array of 13 host-plant species belonging to seven families. Overall, four genetically distinct populations were found. One genetically distinct genotype was exclusively associated with one of the host-plant species while the other three were observed across more than one host-plant species. The relatively low degree of HAD in CFH compared to the pea aphid, another hemipteran insect, stresses the likely importance of sexual recombination as a factor increasing the likelihood of HAD.

## Introduction

Host-plants play an important role in the diversification of insect populations (Ehrlich and Raven 1964). While associated with different host-plant species, insect populations can experience different selection pressures that may create ecological barriers to gene flow (Pashley 1986; Feder et al. 1993; Nosil and Crespi 2006). Divergent selection on different host-plant species may result in adaptive traits responsible for reproductive isolation among host-associated subpopulations. If reproductive isolation is maintained, this process may end up in the formation of genetically distinct host-associated lineages or host races (Diehl and Bush 1984; Bernays 1991; Carroll and Boyd 1992; Pappers et al. 2001; Dres and Mallet 2002). This phenomenon is commonly referred to as host-associated genetic differentiation (HAD) (Bush 1969; Abrahamson et al. 2001).

In recent years, there has been a growing interest in HAD and several studies have sought to investigate the phenomenon in a variety of insect species including specialist (Funk et al. 2002; Althoff et al. 2007; Hernadez-Vera et al. 2010; Heard 2012; Medina et al. 2012) and generalist insects (Dopman et al. 2002; Funk et al. 2002; Sword et al. 2005; Barman et al. 2012). Perhaps some of the best-studied cases of insect HAD are those involving apple maggot flies (*Rhagoletis pomonella*) on apples and hawthorns (Bush 1969; Feder et al. 1993; Forbes et al. 2010), species associated with goldenrods (Abrahamson et al. 2001; Eubanks et al. 2003; Stireman et al. 2005), pea aphids (*Acyrthosiphon pisum*) associated with plants in the Fabaceae family (Via 1999; Frantz et al. 2006; Peccoud et al. 2009), and stick insects (*Timema cristinae)* on redheart and chamise (Nosil et al. 2002; Soria-Carrasco et al. 2014). In all these insect species, genetically distinct lineages have been found on different host-plant species. In fact, the remarkable diversity of insects we see today could be the result of HAD (Ehrlich 1964; Farrell 1998; Abrahamson et al. 2001; Dres and Mallet 2002), making the study of HAD an important component in our understanding of the role of host-plant species in ecological speciation.

Level of intimacy with their hosts (i.e., whether an insect lives/feeds within plant tissues vs. externally) and the type of reproduction (i.e., sexual or asexual) are factors thought to influence the propensity of insects to exhibit HAD (Medina 2012). Much of what we know about HAD involves insect species with narrow host ranges (i.e., specialists) that spend part or all their life cycle inside their hosts, and/or reproduce asexually (e.g., parthenogenetically) (Pashley 1986; Van Zandt and Mopper 1998; Brunner et al. 2004; Dickey and Medina 2010, 2012; Cook et al. 2012; Darwell et al. 2014; Marques et al. 2014). Pea aphids, for example, are parthenogenetic Fabaceae specialists that are composed of genetically distinct host-associated lineages on clover and alfalfa (Via 1999). Even though pea aphids are associated with multiple plant species (Via 1999; Simon et al. 2003; Ferrari et al. 2006; Frantz et al. 2006; Via 2009), it was not until Peccoud et al. (2009) sampled insects from an extensive number of different host-plant populations that HAD in pea aphids was found to be more extensive than previously thought. This raises the question of whether HAD is really uncommon in sexually reproducing generalists or perhaps has simply been overlooked due to limited sampling.

Evidence of HAD in sexually reproducing generalist species is accumulating. For example, grasshoppers and green mirids are polyphagous, feeding on multiple hosts from different families, yet they exhibit HAD (Sword and Dopman 1999; Dopman et al. 2002; Sword et al. 2005; Apple et al. 2010; Hereward et al. 2013). For agriculturally important pests, genetically distinct lineages on different host-plants may differ in their susceptibility to certain pest control methods. Thus, knowing which pest species show HAD is important. For example, conservation biological control may not work in a particular crop if natural enemies co-evolve with their insect hosts on one host-plant species and become reproductively isolated on alternative host plants (Eubanks et al. 2003; Forbes et al. 2010; Heard et al. 2006). Similarly, the use of alternative host-plant species as refuges in transgenic crop plantings may not work if host-associated populations of polyphagous pests are reproductively isolated when on different host-plant species (Calcagno et al. 2007). Although some sexually reproducing generalist pests (e.g., fall armyworm, browntail moth, green mirid) have been shown to exhibit HAD (Pashley 1986; Hereward et al. 2013; Marques et al. 2014), we currently do not know how widespread HAD is across the agroecosystems in which these pest species exist.

The cotton fleahopper (CFH), *Pseudatomoscelis seriatus* Rueter, (Hemiptera: Miridae) offers a good model to test HAD in a sexually reproducing generalist insect pest in a highly managed monoculture. CFH feeds on at least 160 host-plant species belonging to 35 different families of both managed crops and unmanaged wild plants (Snodgrass et al. 1984; Esquivel and Esquivel 2009). It feeds using its piercing–sucking mouthparts on anthers and young flower buds of developing plants. As an agriculturally important crop, cotton (*Gossypium hirsutum*) is most vulnerable to CFH attack during the first three weeks of early flower bud (referred as “squares”) development (Sansone et al. 2009). Recently, Barman et al. (2012) tested for HAD in CFH when associated with three of its most abundant host-plants in Texas, USA: horsemint, *Monarda punctata* L.; woolly croton, *Croton capitatus;* and cotton. CFH on horsemint showed strong HAD in areas where annual precipitation was below 26 inches. Given that CFH is highly polyphagous, we predicted that HAD would be likely to occur on other host-plant species as well. To test this hypothesis, we used AFLP and Bayesian analyses to test for HAD among CFH collected from 13 different host-plant species belonging to seven plant families.

## Materials and Methods

### Cotton fleahopper sampling and host-plant identification

**Figure 1:**
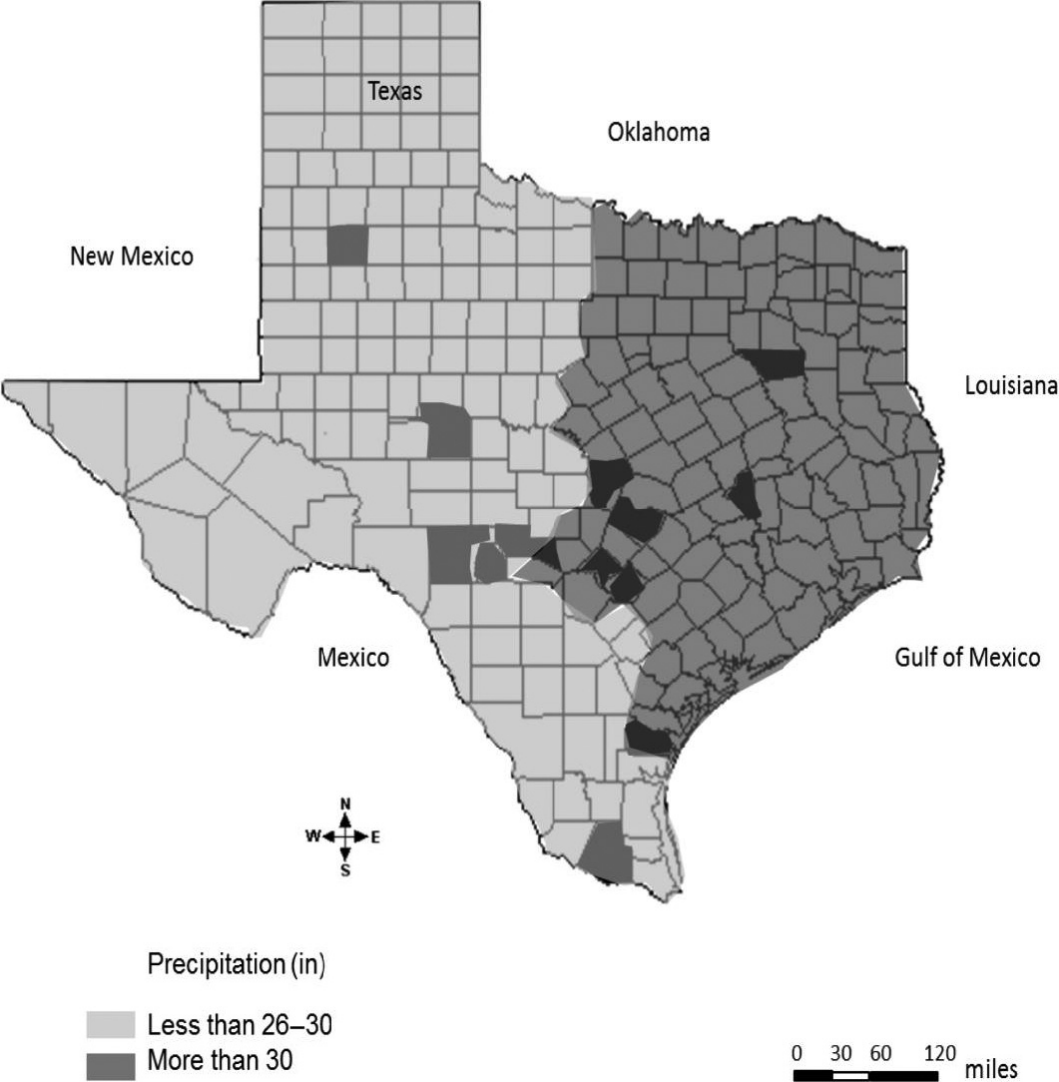
Map of Texas indicating locations where CFH was sampled. Counties where sampling took place are shaded in dark gray black. The entire state is divided into two regions with respect to annual precipitation as described by Barman et al. (2012): regions where annual precipitation is less than 26–30 inches (light gray), and those with precipitation more than 30 inches (medium gray) are indicated on the map.

We sampled CFH from 13 host-plant species (belonging to 7 families), one of which is an annual crop (cotton) and 12 perennial plants (Table1). Plant families sampled included: Asteraceae, Euphorbiaceae, Lamiaceae, Malvaceae, Onagraceae, Solanaceae, and Verbenaceae. CFHs were collected from 14 locations in Texas, spanning 13 counties distributed across multiple ecological regions from the Piney Woods in the east to Edwards Plateau in the west (Fig.1). In addition to collecting CFH individuals, we collected plant samples from which the insects were collected as voucher specimens. Plants were individually pressed using standard plant press protocols (Queensland-Herbarium, 2013). Plants were identified to species by Dr. Dale Kruse (S. M. Tracy Herbarium, Department of Rangeland Ecology and Management, TAMU College Station, Texas). Cotton fleahopper sampling took place during the spring and summer of 2013 and 2014 when herbaceous plants had green foliage, some of which were blooming at the time of sampling. On cotton, CFH sampling coincided with the development of flower buds (“squares”) when CFH numbers were typically high. Using hand-held sweep nets and aspirators, insects were sampled from cotton fields, wild vegetation patches surrounding cotton fields, open fields within natural forest stands, and along roadsides and highways. We initially planned to sample only CFH nymphs from each host plant; however, due to overall low nymph numbers on several of the host plants sampled, we also included adults in this study. In all, a total of 240 individuals were analyzed, ranging from 8 to 20 individuals per host-plant species. Individuals were stored in 80% ethanol prior to DNA extraction.

**Table 1:** Host-plants and sampling locations (counties) in Texas from which CFH individuals were collected. Letters in parenthesis are abbreviations of common names of host plants

Species name	Common name	Family name	Location (County)
*Hymenopappus scabiosaeus*	Old plainsman (OP)	Asteraceae	Travis/Kerr/Burnet
*Ambrosia psilostachya*	Western ragweed (WR)	Asteraceae	Brazos/Nueces
*Oenothera speciosa*	Evening primrose (EP)	Onagraceae	Brazos/Nueces
*Gaura parviflora*	Velvet-leaf beeblossom (VB)	Onagraceae	Brazos
*Gossypium hirsutum*	Cotton (CT)	Malvaceae	Brazos/Nueces/Lubbock/Tom Green/Hildago
*Malvella lepidota*	Scurvy mallow (SM)	Malvaceae	Travis/Burnet
*Croton monanthogynus Michx*.	Oneseed croton (OC)	Euphorbiaceae	Comal
*Croton lindheimerianus Scheele*	Threeseed croton (TC)	Euphorbiaceae	Guadalupe
*Croton argyranthemus Michx*.	Silvercroton (SC)	Euphorbiaceae	Henderson
*Marrubium vulgare L*.	Common horehound (CH)	Lamiaceae	Burnet/Real
*Monarda punctata*	Horsemint (HM)	Lamiaceae	Brazos/Nueces/Lubbock/Tom Green/Hildago
*Solanum elaegnifolium*	Silverleaf nightshade (SN)	Solanaceae	Brazos/Nueces/Lubbock/Burnet
*Glandularia bipinnatifida (Nut.) Nutt*.	Purple praire (PP)	Verbaneceae	Real/Burnet

### DNA extraction and AFLPs

Genomic DNA was extracted from whole insects using DNeasy® tissue extraction kit (QIAGEN, Valencia, CA) following the manufacturer’s protocol and stored in AE buffer at −20°C. DNA concentration and quality were assessed using a NanoDrop spectrophotometer (NanoDrop Technologies, Wilmington, DE). On average, DNA concentration and quality from individual CFH extractions were 100 ng/*μ*L and 2.00, respectively. Amplified fragment length polymorphism (AFLP) reactions were performed following the protocol of Vos et al. (1995) with minor modifications by Barman et al. (2012). Briefly, aliquot of DNA from individuals was randomly assigned to a 96-well plate, repeating one control individual three times on each plate to assess reproducibility. A negative control (blank) was included in every plate to assess potential cross-contamination. A restriction digestion of 5.5 *μ*L DNA and 5.5 *μ*L of master mix containing 0.03 *μ*L T4 DNA ligase (New England Biolabs (NEB), Ipswich, MA), 1.1 *μ*L 10x T4 DNA ligase buffer, 1.1 *μ*L 0.5 mol/L NaCl, 0.55 *μ*L diluted BSA, 0.05 *μ*L MseI (NEB), 0.05 *μ*L EcoRI (NEB), 1 *μ*L each MseI and EcoRI adapter pairs (Life Technologies, Carlsbad, California, USA), and 0.61 *μ*L sterile distilled water was performed. Reactions were incubated overnight after which they were diluted 17-fold, with 189 *μ*L TE_thin_ buffer. This was followed by a 20-*μ*L total volume preselective PCR reaction mix consisting of 4 *μ*L diluted DNA, 15 *μ*L AFLP core mix (Life Technologies), and 1 *μ*L AFLP amplification primers (Life Technologies). Selective PCR amplifications were performed in a 21-*μ*L volume of 15 *μ*L platinum supermix (Life Technologies), 4 *μ*L of a 19-fold diluted pre-amplification reaction product, and one primer combination consisting of 1 *μ*L MseI-CAT (Life Technologies) and 1 *μ*L EcoRI-ACT (Life Technologies). All PCR amplifications were carried out in an ABI GeneAmp thermocycler (Life Technologies) using protocols from Barman et al. (2012). Reactants were prepared in a laminar flow hood. DNA and PCR reagents were added using filter tips to minimize the risk of cross-contamination. A 10.5 *μ*L total volume consisting of 1 *μ*L selective amplification PCR product, 9 *μ*L HiDi formamide, and 0.5 *μ*L ROX 400 size standard (Life Technologies) was used for electrophoretic analysis of selective PCR fragments. Samples were analyzed on an ABI 3730xl 96-capillary genetic analyzer (Applied Biosystems, Forest City, CA).

### Genetic analysis

Amplified fragment length polymorphism fragments were analyzed with the genetic software GeneMarker v.2.6.3 (Softgenetics, State College, Pennsylvania, USA). Only loci with fragment sizes within 50–400 bp and florescent units of 100 or more were included in our analyses. Results from GeneMarker were converted into a binary matrix of presence (1) or absence (0) for each locus. Loci with fewer than 5% markers than the average number of markers per loci were removed from the dataset. Fragment amplification failed in 30 individuals that were accordingly removed from the dataset. To ascertain whether the number of individuals and the number of markers used in the study were sufficient to accurately predict genetic structure of CFH, we used the SESim statistic (Medina et al. 2006). A SESim value lower than 0.05 indicates that the number of loci and individuals in a dataset are sufficient and that additional markers or individuals may not alter the population clustering pattern produced by the sampled area under study (Medina et al. 2006).

Allelic frequencies of AFLP fragments were estimated using the Bayesian method implemented in AFLP-SURV v.1.0 (Vekemans et al. 2002) with the nonuniform prior distributions of allele frequencies option. The Bayesian method of AFLP-SURV produces statistically unbiased estimates of genetic diversity and genetic distances (Zhivotovsky 1999). Allele frequencies were used to estimate overall F_ST_ between host populations and pairwise F_ST_ between host-plants using 100,000 permutations in ARLEQUIN (Excoffier and Lischer 2010). Significance of F_ST_ was estimated with 10,000 permutations. Additionally, Nei’s genetic distances between pairs of populations were estimated in AFLP-SURV using 10,000 permutations. Genetic diversity for each population was measured by estimating the number of polymorphic loci and Nei’s gene diversity.

Genetic distances between pairs of populations were used for principal coordinate analysis (PCoA) in GenAlEx 6.5 (Peakall and Smouse 2012). An analysis of molecular variance (AMOVA) implemented in GenAlEx was also used to estimate hierarchical genetic structure within and among populations using host-plants and geographic location as source populations. Here, sampling locations were grouped by region, that is, east versus west Texas (Fig.1), to reflect the potential effect of precipitation on genetic differentiation as outlined by Barman et al. (2012). We performed a five-part AMOVA: differentiation (1) among host-plants, (2) within host-plants, (3) among sampling regions, (4) among sampling locations within regions, and (5) within sampling locations. AMOVA calculates Φ_PT_, an analogue of *F*_ST_, using a squared Euclidean distance matrix between AFLP fragments. Φ_PT_ is a band-based approach recommended for AFLP data because it does not depend on assumptions that underestimate genetic variability (Lynch and Milligan 1994; Yan et al. 1999). Genetic structure was further assessed with the Bayesian model-based clustering algorithm implemented in STRUCTURE v.2.3.4 (Pritchard et al. 2000; Falush et al. 2007). Clustering in this model was based on the assumption of admixed populations with independent allele frequencies. Sampling source (i.e., host-plant) was used as prior information to assist the clustering method. A burn-in period of 10,000 and a run length of 10,000 Markov chain Monte Carlo (MCMC) iterations were performed for 20 runs for clusters (K) ranging from 1 to 14. Delta K (ΔK) was estimated based on Evanno et al. (2005) to select K with the highest probability of predicting population structure in the dataset. Given that populations with stronger structuring may hide structuring in other populations (Evanno et al. 2005), STRUCTURE was first run on the whole dataset, then rerun after consecutively removing populations with distinct clusters (i.e., scurvy mallow and horsemint) (Figures S1–S3).

## Results

The primer pair used in this study yielded a total of 62 AFLP loci. A SESim statistic of 0.011 indicated that the number of loci and individuals in our dataset were sufficient to describe the population clustering pattern produced by CFH in our study (Medina et al. 2006). The percentage of polymorphic loci per host-plant ranged from 45% to 79% with scurvy mallow (SM) and both cotton (CT) and primrose (EP) yielding the lowest and highest polymorphisms, respectively (Table2). Estimates of Nei’s genetic diversity were similar across host-plants with an average of 0.06 (SE = 0.003). Overall, genetic differentiation of host-plants based on *F*_ST_ was low, but significant (0.07; *P *=* *0.01). Pairwise *F*_ST_ values among host-plants indicate that genetic differentiation was either absent or low among most hosts (Table3). CFHs on scurvy mallow (SM), however, were genetically distinct when compared with all other hosts, with pairwise F_ST_ estimates ranging from 0.29 to 0.32. Likewise, differentiation of horsemint (HM) was significantly different from other hosts, with pairwise F_ST_ ranging from 0.06 to 0.16 (Table3).

**Table 2:** Genetic diversity indices of CFH collected from different host-plants based on AFLP data. Host-plants are abbreviated by their common names (see Table1 for taxonomic information)

Host plant	*N* 1	PLP^2^	Hj^3^ (SE)
OP	19	72.6	0.15 (0.01)
WR	19	75.8	0.17 (0.01)
EP	20	79	0.18 (0.01)
VB	18	73	0.17 (0.01)
CT	14	79	0.14 (0.01)
SM	18	45	0.18 (0.02)
OC	20	77	0.17 (0.01)
TC	19	68	0.12 (0.01)
SC	8	66	0.17 (0.02)
CH	16	66	0.18 (0.01)
HM	19	53	0.13 (0.01)
SN	18	65	0.13 (0.01)
PP	23	74	0.15 (0.01)

1 Number of samples.

2 Proportion of polymorphic loci at the 5% level.

3 Expected heterozygosity under Hardy–Weinberg genotypic proportions (or Nei’s gene diversity).

Total *F*_ST_ = 0.03.

**Table 3:** Pairwise F_ST_ estimates of host-associated CFH populations. Host-plants are abbreviated by their common names (see Table1 for taxonomic information). Values in bold represent significantly different *F*_ST_ estimates at 0.05 significance level

Host-plant	CT	TC	VB	CH	HM	SN	OC	OP	EP	PP	WR	SM	SC
CT	–												
TC	0.02	–											
VB	0.04	0.05	–										
CH	0.05	0.06	0.05	–									
HM	**0.08**	**0.09**	**0.09**	**0.11**	–								
SN	0.03	0.01	**0.06**	**0.06**	**0.09**	–							
OC	0.02	0.04	0.03	0.03	**0.10**	0.05	–						
OP	0.02	0.02	0.04	0.02	**0.06**	0.02	0.02	–					
EP	0.03	0.02	0.02	0.02	**0.08**	0.03	0.02	0.01	–				
PP	0.04	0.03	**0.05**	0.04	**0.09**	0.03	0.02	0.02	0.02	–			
WR	0.04	0.04	0.04	0.02	**0.10**	**0.06**	0.03	0.02	0.01	0.04	–		
SM	**0.32**	**0.30**	**0.29**	**0.32**	**0.30**	**0.30**	**0.30**	**0.30**	**0.29**	**0.31**	**0.30**	–	
SC	0.08	**0.10**	0.06	0.05	**0.16**	**0.11**	0.05	0.06	0.05	**0.08**	0.08	**0.35**	–

When host-plants from all locations were grouped together in the AMOVAs, genetic differentiation among host plants explained low but significant variation in CFH (7%), whereas much of the variation was explained within host-plants (i.e., 93%). When sampling locations were grouped by region (i.e., east vs. west Texas [Fig.1]) to reflect the potential effects of precipitation on genetic differentiation (Barman et al. 2012), AMOVA detected 96% variation within locations while variation among regions and variation among locations within regions explained only 0% and 3%, respectively (Table4).

**Table 4:** AMOVA results for CFH populations indicating the amount of variation accounted for (a) among host-plants, (b) within host-plants, (c) among regions considering east versus west Texas as distinct regions, (d) among counties in west and east Texas, and (e) within locations sample in each county

Source of variation	df	SS	Estimated variance	Percent (%) variation	Φ statistic	*P* value
Host-plants						
(a) Among host-plants	12	232.21	0.72	8	PT = 0.08	0.01
(b) Within host-plants	188	1541.20	8.20	92		
Locations						
(c) Among regions	1	15.51	0.03	0	RT = 0.00	0.14
(d) Among locations within region	9	122.11	0.28	3	PR = 0.03	0.00
(e) Within locations	188	1614.30	8.59	96	PT = 0.04	0.00

**Figure 2:**
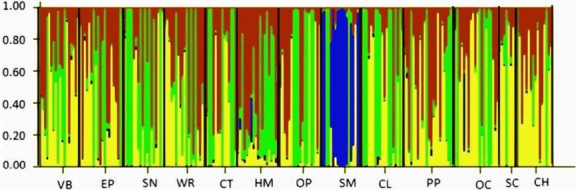
Structure output when ΔK = 4 for both and adults and nymphs of CFH associated with 13 host-plants. Host-plants abbreviated by their common names (see Table1) are indicated below and separated by black bars. Each colored bar represents an individual CFH with the proportion of color corresponding to the probability that an individual is a member of a particular cluster.

**Figure 3:**
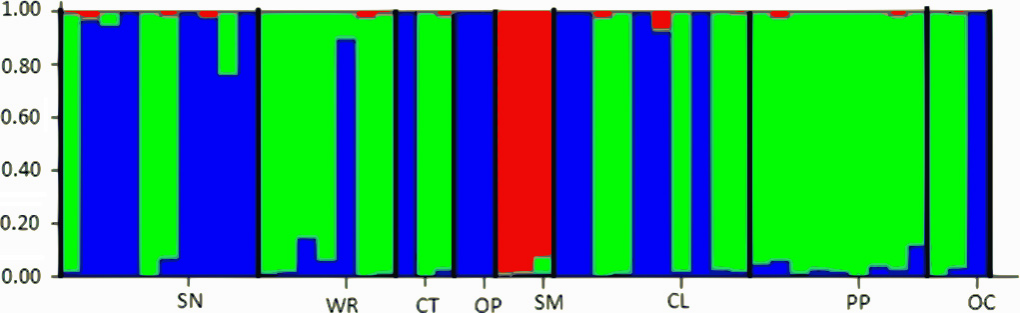
Structure output when ΔK = 3 for CFH nymphs associated with 8 host plants. Host plants abbreviated by their common names (see Table1) are indicated below and separated by black bars. Each colored bar represents an individual CFH with the proportion of color corresponding to the probability that an individual is a member of a particular cluster.

**Figure 4:**
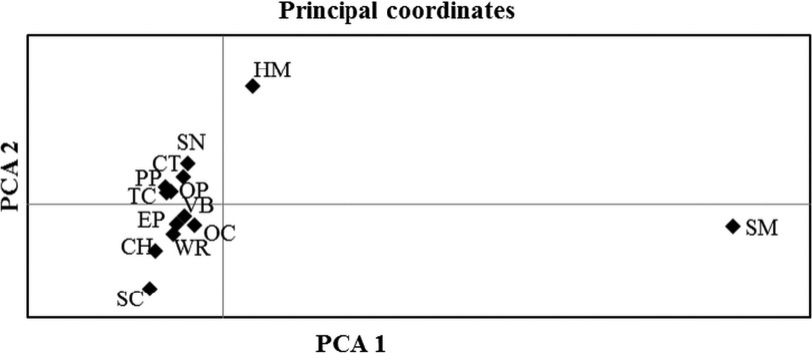
Principal coordinates 1 (*x*-axis) and 2 (*y*-axis) for CFH associated with 13 host-plants. Host-plants are abbreviated by their common names and denoted by filled diamonds. PCA1 explains 63.8% of the variation; PCA2 explains 18.8%.

Genetic structure was further investigated with the Bayesian-based clustering algorithm in STRUCTURE. Using the complete dataset with individuals from all host plants across sampling locations, ΔK (Evanno et al. 2005) detected 4 genetically distinct genetic origins (Fig.2). In agreement with our other analyses, the scurvy mallow cluster was genetically distinct from individuals collected from all other hosts, with a high probability (approximately >99%) of individual assignment. Individuals from the other host-plants displayed a mixed genotype that varied in relative composition on different host-plant species (Fig.2). A separate analysis in STRUCTURE using only nymphs (from 8 host-plants on which nymphs were sampled) also differentiated CFH on scurvy mallow from those collected from other hosts. However, ΔK for nymphs indicated only three genetic origins (Fig.3). Interestingly, nymphs from purple prairie (PP) belonged to only one genetically distinct population while adults on this plant belonged to three populations. Finally, principal coordinate analyses (PCoAs) 1 and 2 explained 82.6% of the genetic variation of CFH (Fig.4). PCoA 1 separated CFHs from scurvy mallow (SM) and horsemint (HM) from CFHs collected from every other host, whereas PCoA 2 separated only CFH from horsemint.

## Discussion

Given that HAD is known to occur in the CFH (Barman et al. 2012) and that it has an extensive host-plant range of over 160 plants, we predicted that expanded sampling for HAD would reveal additional instances of HAD. Our results provided limited support for our hypothesis. CFH exhibited slight, but significant, genetic structuring across multiple host-plant species. Although estimates of genetic differentiation were low, host-plants explained a higher proportion of genetic variation in CFH than geographic location (Table4). The first study of HAD in the CFH by Barman et al. (2012) included only three host-plant species. In this study, we expanded the assessment of HAD by analyzing CFHs collected from 13 host-plant species. Results from our study identified 4 genetically distinct populations of CFH associated with 13 host-plant species (Fig.2), of which two were distinctly associated with a specific host-plant. CFHs from scurvy mallow were genetically distinct from CFHs from any other host (Figs.4; Table3). CFHs collected from horsemint were also differentiated when compared with CFHs collected from other host-plants (Fig.4 and Table3). However, CFH genotypes on horsemint, although differentiated, were not unique to horsemint (Fig.2).

In their study, Barman et al. (2012) found that CFH associated with horsemint showed strong HAD but the pattern of differentiation was typical of a “geographic mosaic of HAD”. In other words, horsemint populations in west Texas displayed a strong pattern of HAD, but in east Texas HAD was absent. Barman et al. (2012) speculated that the patchy distribution of horsemint in west Texas relative to the plant’s almost continuous distribution in east Texas could potentially explain the differential presence of HAD in these two regions. Our study examined CFH in west Texas populations not only on horsemint and cotton (as in the Barman et al. 2012 study), but also on other uncultivated plants (e.g., silverleaf nightshade, purple prairie, old plainsman, and common horehound), revealing that CFH genotypes found on horsemint were not uniquely associated with this plant species. That is, the horsemint genotype characterized by Barman et al. (2012) was also present in other uncultivated plant species. Both Barman et al. (2012) and this study examined adults on horsemint. However, future studies should genetically characterize nymphs (see below for further discussion about nymphs) on horsemint and compare them with nymphs from uncultivated vegetation. Comparing genetic population structure of nymphs versus adults from different host-plant species will increase our understanding of CFH host-plant fidelity, mating, and dispersal behavior.

Populations on scurvy mallow were genetically distinct from those on the other hosts tested in this study. The scurvy mallow plants we sampled were in close proximity with old plainsman, silverleaf nightshade, common horehound, and purple prairie. In order for host-related selection to cause divergence among populations in such close proximity, there has to be sufficient reduction in gene flow among populations associated with these different host-plants (Geiselhardt et al. 2012). If divergent selection on CFH associated with scurvy mallow is linked to mating and/or oviposition preference, then selection may have favored assortative mating on scurvy mallow facilitating HAD.

In pea aphids, HAD was first reported in populations associated with alfalfa and red clover (Via 1999; Leonardo and Muiru 2003). Later, another distinct lineage of pea aphids was found on populations associated with pea and faba bean (Carre and Bournoville 2003; Simon et al. 2003; Frantz et al. 2006). After testing for HAD on 19 widely distributed plants, Peccoud et al. (2009) found 11 distinct host-associated lineages of pea aphids in Western Europe. In the case of the highly polyphagous CFH, when Barman et al. (2012) tested HAD on three host-plants, they detected one host-associated lineage. In our study, extending the number of host plants did not dramatically increase the incidence of HAD, suggesting that compared to pea aphids, HAD is rather uncommon in CFH. The scarcity of HAD in CFH compared to pea aphids may at least partly be explained by differences in their mode of reproduction.

It has been proposed that parthenogenesis may increase the incidence of HAD (Medina 2012). In fact, several HAD case studies involve parthenogenetic organisms such as pea aphids (Via 1999), grain aphids (Simon et al. 1999; Vialatte et al. 2005), yellow pecan aphids (Dickey and Medina 2010), western flower thrips (Brunner et al. 2004; Brunner and Frey 2010), and eriophyid mites (Evans et al. 2013). However, HAD also occurs in sexually reproducing insects such as grasshoppers (Dopman et al. 2002; Sword et al. 2005; Apple et al. 2010), green mirids (Hereward et al. 2013), fall armyworms (Pashley 1986), and brown tail moths (Marques et al. 2014). Unfortunately, in all these cases HAD was tested across only a handful of host-plant species, making it impossible to know whether HAD extends beyond the sampled plants. We predict that HAD in sexually reproducing insect herbivores will parallel the pattern we have found in the CFH. That is, HAD will be present in a rather small proportion of host-plants. On the contrary, HAD in parthenogenetic herbivores is expected to be present in several of their host-plants, as it has already been reported in the pea and cotton aphids (Vanlerberghe-Masutti and Chavigny 1998; Ferrari et al. 2006; Peccoud et al. 2009).

To test whether dispersing adults were confounding the population structure found in this study, we used only nymphs (due to their relatively low dispersibility) in a separate STRUCTURE analysis. Although the analysis of nymphs did not dramatically change the overall pattern of HAD in CFH (Fig.3), it made it less “noisy”. Interestingly, nymphs on purple prairie harbored only one genotype (Fig.2) while adult populations harbored three (Fig.2). All other plants, except for scurvy mallow, supported two genotypes when only nymphs were considered (Fig.3). The differences observed in the analyses of nymph and adult genetic population structure could be explained by adult dispersal among host-plant species.

Host-associated differentiation of CFH populations may have practical implications for pest control. The fact that genotypes found in cotton can also be found in nearby uncultivated vegetation suggests that several native hosts-plants act as sources of CFH in cotton fields. However, some host-plant species such as scurvy mallow and horsemint harbor CFH genotypes that are genetically distinct and may not contribute to building up pestiferous populations in cotton. This same phenomenon has been observed in wheat where populations of cereal aphid, *Sitobion avenae*, associated with wild vegetation do not contribute to the buildup of pestiferous populations in wheat (Vialatte et al. 2005). Thus, plants such as scurvy mallow and horsemint could be considered as plants suitable to use in conservation biological control programs to enhance local CFH natural enemy populations. Interestingly, CFH populations in horsemint have been found to be genetically distinct only in west Texas. Populations of CFH in east Texas are identical to CFH populations in cotton (Barman et al. 2012). Geographic variation in the pattern of HAD stresses the need to study pests’ population structure across their entire geographic distribution and host range. Genetic population structure of pest species may inform locally adapted control strategies in area-wide integrated pest management (IPM) programs.

## Supporting Information

**Figure S1.** Structure output when individuals from scurvy mallow (SM) were removed from the analysis. **Figure S2.** Structure output when individuals from horsemint (HM) were removed from the analysis. **Figure S3.** Structure output when individuals from horsemint (HM) and scurvy mallow (SM) were removed from the analysis. **Figure S4.** Cotton fleahopper feeding on one its wild host-plants.

## References

[b1] Abrahamson WG, Eubanks MD, Blair CP, Whipple AV (2001). Gall flies, inquilines, and goldenrods: a model for host-race formation and sympatric speciation. Am. Zool.

[b2] Althoff DM, Svensson GP, Pellmyr O (2007). The influence of interaction type and feeding location on the phylogeographic structure of the yucca moth community associated with Hesperoyucca whipplei. Mol. Phylogenet. Evol.

[b3] Apple JL, Grace T, Joern A, Amand PS, Wisely SM (2010). Comparative genome scan detects host-related divergent selection in the grasshopper *Hesperotettix viridis*. Mol. Ecol.

[b4] Barman AK, Parajulee MN, Sansone CG, Suh CPC, Medina RF (2012). Geographic pattern of host-associated differentiation in the cotton fleahopper, *Pseudatomoscelis seriatus*. Entomol. Exp. Appl.

[b6] Bernays EA (1991). Evolution of insect morphology in relation to plants. Philos. Trans. R. Soc. Lond. B Biol. Sci.

[b8] Brunner PC, Frey JE (2010). Habitat-specific population structure in native western flower thrips *Frankliniella occidentalis* (Insecta, Thysanoptera). J. Evol. Biol.

[b9] Brunner PC, Chatzivassiliou EK, Katis NI, Frey JE (2004). Host-associated genetic differentiation in *Thrips tabaci* (Insecta; Thysanoptera), as determined from mtDNA sequence data. Heredity.

[b10] Bush GL (1969). Sympatric host race formation and speciation in frugivorous flies of genus *Rhagoletis* (Diptera, Tephritidae). Evolution.

[b11] Calcagno V, Thomas Y, Bourguet D (2007). Sympatric host races of the European corn borer: adaptation to host plants and hybrid performance. J. Evol. Biol.

[b12] Carre S, Bournoville R (2003). Specialization of spring sympatric populations of *Acyrthosiphon pisum* (Hemiptera: Aphididae) according to Fabaceae. Annu. Soc. Entomol. Fr.

[b13] Carroll SP, Boyd C (1992). Host race radiation in the soapberry bug – natural history with the history. Evolution.

[b14] Cook MA, Fitzpatrick SM, Roitberg BD (2012). Phenology of *Dasineura oxycoccana* (Diptera: Cecidomyiidae) on cranberry and blueberry indicates potential for gene flow. J. Econ. Entomol.

[b15] Darwell CT, Fox KA, Althoff DM (2014). The roles of geography and founder effects in promoting host-associated differentiation in the generalist bogus yucca moth *Prodoxus decipiens*. J. Evol. Biol.

[b16] Dickey AM, Medina RF (2010). Testing host-associated differentiation in a quasi-endophage and a parthenogen on native trees. J. Evol. Biol.

[b17] Dickey AM, Medina RF (2012). Host-associated genetic differentiation in pecan leaf phylloxera. Entomol. Exp. Appl.

[b18] Diehl SR, Bush GL (1984). An evolutionary and applied perspective of insect biotypes. Annu. Rev. Entomol.

[b19] Dopman EB, Sword GA, Hillis DM (2002). The importance of the ontogenetic niche in resource-associated divergence: evidence from a generalist grasshopper. Evolution.

[b20] Dres M, Mallet J (2002). Host races in plant-feeding insects and their importance in sympatric speciation. Philos. Trans. R. Soc. Lond. B Biol. Sci.

[b23] Ehrlich PR, Raven PH (1964). Butterflies and plants – a study in coevolution. Evolution.

[b24] Esquivel JF, Esquivel SV (2009). Identification of cotton fleahopper (Hemiptera: Miridae) host plants in central Texas and compendium of reported hosts in the United States. Environ. Entomol.

[b25] Eubanks MD, Blair CP, Abrahamson WG (2003). One host shift leads to another? Evidence of host-race formation in a predaceous gall-boring beetle. Evolution.

[b26] Evanno G, Regnaut S, Goudet J (2005). Detecting the number of clusters of individuals using the software STRUCTURE: a simulation study. Mol. Ecol.

[b27] Evans LM, Allan GJ, Meneses N, Max TL, Whitham TG (2013). Herbivore host-associated genetic differentiation depends on the scale of plant genetic variation examined. Evol. Ecol.

[b28] Excoffier L, Lischer HEL (2010). Arlequin suite ver 3.5: a new series of programs to perform population genetics analyses under Linux and Windows. Mol. Ecol. Res.

[b29] Falush D, Stephens M, Pritchard JK (2007). Inference of population structure using multilocus genotype data: dominant markers and null alleles. Mol. Ecol. Notes.

[b30] Farrell BD (1998). “Inordinate fondness” explained: why are there so many beetles?. Science.

[b31] Feder JL, Hunt TA, Bush GL (1993). The effects of climate, host-plant phenology and host fidelity on the genetics of apple and hawthorn infesting races of *Rhagoletis Pomonella*. Entomol. Exp. Appl.

[b32] Ferrari J, Godfray HCJ, Faulconbridge AS, Prior K, Via S (2006). Population differentiation and genetic variation in host choice among pea aphids from eight host plant genera. Evolution.

[b33] Forbes AA, Hood CR, Feder JL (2010). Geographic and ecological overlap of parasitoid wasps associated with the *Rhagoletis pomonella* (Diptera: Tephritidae) species complex. Ann. Entomol. Soc. Am.

[b34] Frantz A, Plantegenest M, Mieuzet L, Simon JC (2006). Ecological specialization correlates with genotypic differentiation in sympatric host-populations of the pea aphid. J. Evol. Biol.

[b35] Funk DJ, Filchak KE, Feder JL (2002). Herbivorous insects: model systems for the comparative study of speciation ecology. Genetica.

[b36] Geiselhardt S, Otte T, Hilker M (2012). Looking for a similar partner: host plants shape mating preferences of herbivorous insects by altering their contact pheromones. Ecol. Lett.

[b37] Heard SB (2012). Use of Host-plant trait space by phytophagous insects during host-associated differentiation: the gape-and-pinch model. Intl. J. Ecol.

[b38] Heard SB, Stireman JO, Nason JD, Cox GH, Kolacz CR, Brown JM (2006). On the elusiveness of enemy-free space: spatial, temporal, and host-plant-related variation in parasitoid attack rates on three gallmakers of goldenrods. Oecologia.

[b39] Hereward JP, Walter GH, DeBarro PJ, Lowe AJ, Riginos C (2013). Gene flow in the green mirid, *Creontiades dilutus* (Hemiptera: Miridae), across arid and agricultural environments with different host plant species. Ecol. Evol.

[b40] Hernadez-Vera G, Mitrovic M, Jovic J, Tosevski IVO, Caldara R, Gassmann A (2010). Host-associated genetic differentiation in a seed parasitic weevil *Rhinusa antirrhini* (Coleptera: Curculionidae) revealed by mitochondrial and nuclear sequence data. Mol. Ecol.

[b41] Leonardo TE, Muiru GT (2003). Facultative symbionts are associated with host plant specialization in pea aphid populations. Proc. R. Soc. Lond. B Biol. Sci.

[b42] Lynch M, Milligan BG (1994). Analysis of population genetic structure with Rapd markers. Mol. Ecol.

[b43] Marques JF, Wang HL, Svensson GP, Frago E, Anderbrant O (2014). Genetic divergence and evidence for sympatric host-races in the highly polyphagous brown tail moth, *Euproctis chrysorrhoea* (Lepidoptera: Erebidae). Evol. Ecol.

[b44] Medina RF, Barbosa P, Letourneau DK, Agrawal A (2012). Implications of host-associated differentiation in the control of pest species. Insect outbreaks revisited.

[b45] Medina RF, Barbosa P, Christman M, Battisti A (2006). Number of individuals and molecular markers to use in genetic differentiation studies. Mol. Ecol. Notes.

[b46] Medina RF, Reyna SM, Bernal JS (2012). Population genetic structure of a specialist leafhopper on Zea: likely anthropogenic and ecological determinants of gene flow. Entomol. Exp. Appl.

[b47] Nosil P, Crespi BJ (2006). Ecological divergence promotes the evolution of cryptic reproductive isolation. Proc. R. Soc. Lond. B Biol. Sci.

[b48] Nosil P, Crespi BJ, Sandoval CP (2002). Host-plant adaptation drives the parallel evolution of reproductive isolation. Nature.

[b49] Pappers SM, van Dommelen H, van der Velde G, Ouborg NJ (2001). Differences in morphology and reproductive traits of Galerucella nymphaeae from four host plant species. Entomol. Exp. App.

[b50] Pashley DP (1986). Host-associated genetic differentiation in fall armyworm (Lepidoptera, Noctuidae) – a sibling species complex. Ann. Entomol. Soc. Am.

[b51] Peakall R, Smouse PE (2012). GenAlEx 6.5: genetic analysis in Excel. Population genetic software for teaching and research-an update. Bioinformatics.

[b52] Peccoud J, Ollivier A, Plantegenest M, Simon J-C (2009). A continuum of genetic divergence from sympatric host races to species in the pea aphid complex. Proc. Natl Acad. Sci. USA.

[b53] Pritchard JK, Stephens M, Donnelly P (2000). Inference of population structure using multilocus genotype data. Genetics.

[b54] Queensland-Herbarium (2013).

[b55] Sansone CG, Parajulee MN, Minsenmayer RR, Suh C, Barman AK, Medina RF (2009). Investigations into timing and frequency of insecticide applications for cotton fleahopper. National Cotton Concil of America.

[b56] Simon JC, Baumann S, Sunnucks P, Hebert PDN, Pierre JS, Le Gallic JF (1999). Reproductive mode and population genetic structure of the cereal aphid *Sitobion avenae* studied using phenotypic and microsatellite markers. Mol. Ecol.

[b57] Simon JC, Carre S, Boutin M, Prunier-Leterme N, Sabater-Munoz B, Latorre A (2003). Host-based divergence in populations of the pea aphid: insights from nuclear markers and the prevalence of facultative symbionts. Proc. R. Soc. B Biol. Sci.

[b58] Snodgrass GL, Scott WP, Smith JW (1984). A survey of the host plants and seasonal distribution of the cotton fleahopper (Hemiptera: Miridae) in the Delta of Arkansas, Louisiana, and Mississippi. J. Georgia Entomol. Sci.

[b59] Soria-Carrasco V, Gompert Z, Comeault AA, Farkas TE, Parchman TL, Johnston JS (2014). Stick insect genomes reveal natural selection’s role in parallel speciation. Science.

[b60] Stireman JO, Nason JD, Heard SB (2005). Host-associated genetic differentiation in phytophagous insects: general phenomenon or isolated exceptions? Evidence from a goldenrod-insect community. Evolution.

[b61] Sword GA, Dopman EB (1999). Developmental specialization and geographic structure of host plant use in a polyphagous grasshopper, *Schistocerca emarginata* (lineata) (Orthoptera: Acrididae). Oecologia.

[b62] Sword GA, Joern A, Senior LB (2005). Host plant-associated genetic differentiation in the snakeweed grasshopper, Hesperotettix viridis (Orthoptera: Acrididae). Mol. Ecol.

[b63] Van Zandt PA, Mopper S (1998). A meta-analysis of adaptive deme formation in phytophagous insect populations. Am. Nat.

[b64] Vanlerberghe-Masutti F, Chavigny P (1998). Host-based genetic differentiation in the aphid *Aphis gossypii* Glover, evidenced from RAPD fingerprints. Mol. Ecol.

[b65] Vekemans X, Beauwens T, Lemaire M, Roldan-Ruiz I (2002). Data from amplified fragment length polymorphism (AFLP) markers show indication of size homoplasy and of a relationship between degree of homoplasy and fragment size. Mol. Ecol.

[b67] Via S (1999). Reproductive isolation between sympatric races of pea aphids. I. gene flow restriction and habitat choice. Evolution.

[b68] Via S (2009). Natural selection in action during speciation. Proc. Natl Acad. Sci. USA.

[b69] Vialatte A, Dedryver CA, Simon JC, Galman M, Plantegenest M (2005). Limited genetic exchanges between populations of an insect pest living on uncultivated and related cultivated host plants. Proc. R. Soc. B Biol. Sci.

[b70] Vos P, Hogers R, Bleeker M, Reijans M, Lee T, Hornes M (1995). AFLP: a new technique for DNA fingerprinting. Nucl. Aci. Res.

[b71] Yan G, Romero-Severson J, Walton M, Chadee DD, Severson DW (1999). Population genetics of the yellow fever mosquito in Trinidad: comparisons of amplified fragment length polymorphism (AFLP) and restriction fragment length polymorphism (RFLP) markers. Mol. Ecol.

[b72] Zhivotovsky LA (1999). Estimating population structure in diploids with multilocus dominant DNA markers. Mol. Ecol.

