# Supplementary material for: Host-associated differentiation in a highly polyphagous, sexually reproducing insect herbivore
Source: Ecol Evol. 2015 Jun 1;5(13):2533–43. doi: 10.1002/ece3.1526 (PMC4523351; doi:10.1002/ece3.1526)
Supplement: Supplementary file 1 [file ece30005-2533-sd1.docx]

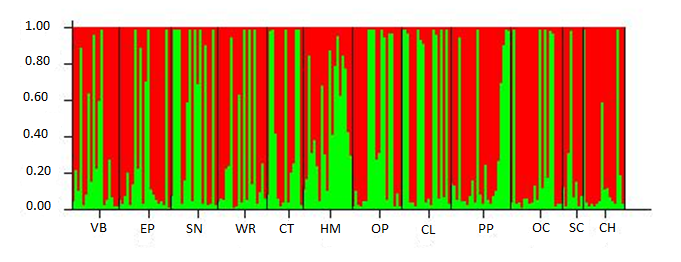
Figure S1. Structure output when individuals from scurvy mallow (SM) were removed from the analysis. Individuals include both adults and nymphs of CFH collected from 12 host plants. Host-plants abbreviated by their common names (see table 1) are indicated below and separated by black bars. Each colored line represents an individual CFH with the proportion of color corresponding to the probability that an individual is a member of a particular cluster.


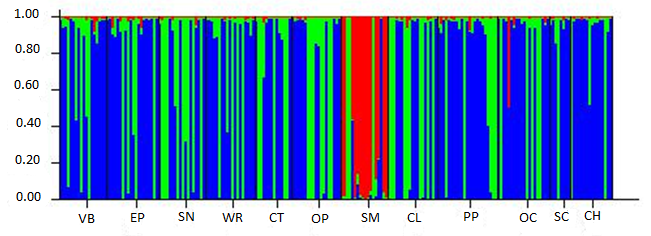
Figure S2. Structure output when individuals from horsemint (HM) were removed from the analysis. Individuals include both adults and nymphs of CFH collected from 12 host plants. Host-plants abbreviated by their common names (see table 1) are indicated below and separated by black bars. Each colored line represents an individual CFH with the proportion of color corresponding to the probability that an individual is a member of a particular cluster.


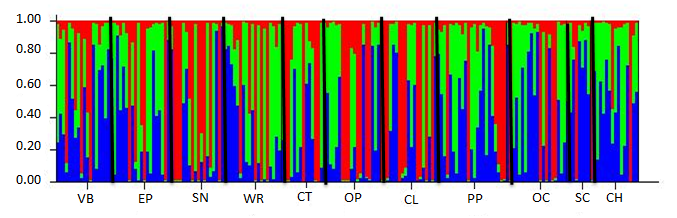
Figure S3. Structure output when individuals from horsemint (HM) and scurvy mallow (SM) were removed from the analysis. Individuals include both adults and nymphs of CFH collected from 11 host plants. Host-plants abbreviated by their common names (see table 1) are indicated below and separated by black bars. Each colored line represents an individual CFH with the proportion of color corresponding to the probability that an individual is a member of a particular cluster.


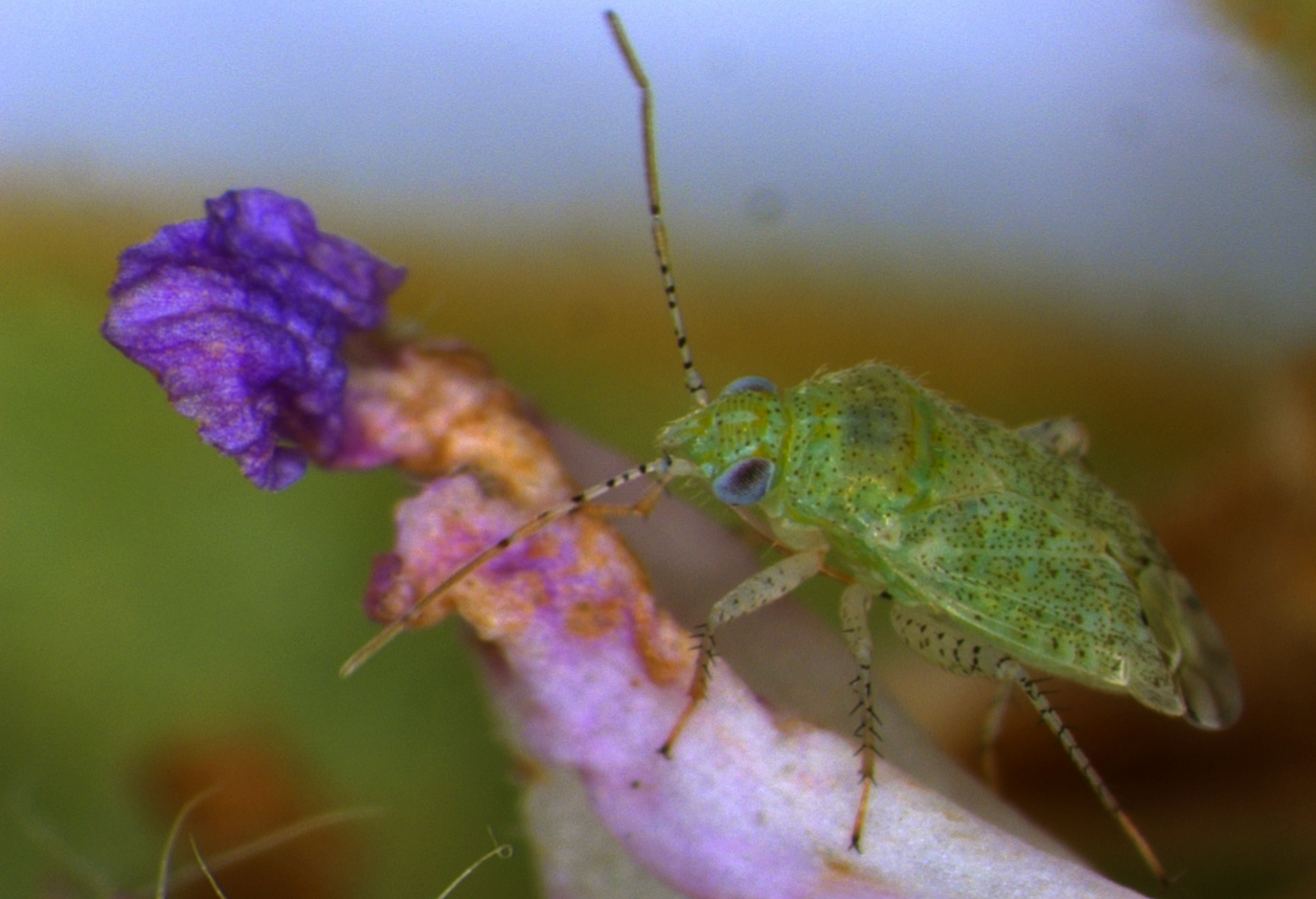


Figure S4. Cotton fleahopper feeding on one its wild host-plants. Photo taken by Apurba Barman. Photo intended for consideration only for publication cover.
